# Variability in social reasoning: the influence of attachment security on the attribution of goals

**DOI:** 10.3389/fpsyg.2015.01487

**Published:** 2015-10-09

**Authors:** Kristen A. Dunfield, Susan C. Johnson

**Affiliations:** ^1^Department of Psychology, Center for Research in Human Development, Concordia University, Montreal, QC, Canada; ^2^Department of Psychology, The Ohio State University, Columbus, OH, USA

**Keywords:** social-cognitive development, social development, cognitive development, social evaluation, attachment, prosocial behavior

## Abstract

Over the last half decade there has been a growing move to apply the methods and theory of cognitive development to questions regarding infants’ social understanding. Though this combination has afforded exciting opportunities to better understand our species’ unique social cognitive abilities, the resulting findings do not always lead to the same conclusions. For example, a growing body of research has found support for both universal similarity and individual differences in infants’ social reasoning about others’ responses to incomplete goals. The present research examines this apparent contradiction by assessing the influence of attachment security on the ability of university undergraduates to represent instrumental needs versus social-emotional distress. When the two varieties of goals were clearly differentiated, we observed a universally similar pattern of results (Experiments 1A/B). However, when the goals were combined, and both instrumental need and social-emotional distress were presented together, individual differences emerged (Experiments 2 and 3). Taken together, these results demonstrate that by integrating the two perspectives of shared universals and individual differences, important points of contact can be revealed supporting a deeper, more nuanced understanding of the nature of human social reasoning.

## Introduction

Humans are unique in the integral role that social relationships play in our success as a species (e.g., Brewer and Caporael, 2006). As a result, there is considerable interest in understanding how individuals come to understand, engage with, and navigate their social environment. Though historically social development and cognitive development were viewed as integrally intertwined (e.g., Piaget, 1945/1995; Vygotsky, 1978; see also, Dweck, 2013), for decades these two lines of inquiry have been pursued largely independently; with social developmentalists typically examining how variability in experiences leads to differences in well-being while cognitive developmentalists typically examine commonalities in the content and development of children’s minds. Recently these two perspectives have been reunited (e.g.,Olson and Dweck, 2008, 2009; Dweck, 2013). This integration has helped build important points of contact between a variety of sub-disciplines of psychology, however in doing so, it has become apparent that sometimes studies which appear to address highly similar questions may lead to quite different conclusions.

### Variability in the Consistency of Social Reasoning

One area where similar studies have led to different conclusions is within the domain of social reasoning. Specifically, a recent explosion of interest in children’s reasoning about others—particularly within the domain of other-oriented behavior—has led to a body of literature in which infants’ representation of positive versus negative interactions (e.g., Premack and Premack, 1997), preferences for helpers versus hinderers (e.g., Hamlin et al., 2007), and expectations following prosocial versus antisocial interactions (e.g., Kuhlmeier et al., 2003; Johnson et al., 2007) appear to support both universal consistency and individual differences (e.g., Johnson et al., 2013).

#### Universal Expectations of Helpers and Hinderers

One line of research utilizes the “helper/hinderer paradigm” to examine infants’ reasoning about others’ responses to instrumental needs and finds a single pattern of common expectations. In these studies, infants watch a brief animation of small ball (the “Climber”) trying and failing to reach the top of a steep hill. On alternating trials, one of two similarly sized shapes (typically a triangle and square) comes down and either pushes the Climber to the top of the hill (the “Helper”) or pushes the Climber to the bottom of the hill (the “Hinderer”). Across a variety of dependent measures, infants appear surprisingly consistent in their expectations of, and preferences for, helpful versus hindering characters.

In the original version of the helper/hinderer paradigm, after infants were habituated to the climb, they were shown the three characters interacting in a novel context. By 12 months, infants differentiated between scenes in which the Climber approached the Helper versus the Hinderer and preferred the video in which the Climber approached the Helper (Kuhlmeier et al., 2003). This preference was consistent with pilot adult participants’ tendency to report seeing “the ball as ‘liking’ or ‘preferring’ the helper object” (Kuhlmeier et al., 2003, p. 402). And, although the participants varied in the degree to which they differentiated between the two types of approach, infants who showed the largest difference in attention to the generally preferred (approach Helper) over non-preferred (approach Hinderer) outcome showed more advanced theory of mind at 4 years than infants who show smaller, or reversed, differences in attention (Yamaguchi et al., 2009); suggesting that this preference was not only shared across individuals but was also associated with relatively more mature social cognitive development.

More recent research finds that infants not only differentiate between these two varieties of approach, but also actively predict them. Using eye-tracking methodology, 12-month-old infants’ anticipatory looks were recorded while they observed the Climber ambiguously approaching the Helper or Hinderer. Twelve out of 17 infants (70.5%) predicted that the Climber would approach the Helper as opposed to the Hinderer (Fawcett and Liszkowski, 2012). Moreover, when given the opportunity to choose between the Helper and Hinderer, 12 out of 12 (100%) 6-month-olds and 14 out of 16 (87.5%) 10-month-olds preferred the Helper (Experiment 1, Hamlin et al., 2007; see also Hamlin, 2014 for a replication of this finding). Together, these studies converge to suggest that when evaluating others’ responses to instrumental needs, most infants prefer helpers to hinderers and expect others to feel similarly. Indeed, these results are so striking that they have been used as evidence in support of the existence of a universal, innate moral core (Hamlin, 2013).

#### Individual Differences in Expectations of Caregivers

In contrast, when infants’ reasoning about others’ responses to social emotional distress have been investigated utilizing a “caregiver paradigm”, the results appear to support robust individual differences in expectations (Johnson et al., 2007, 2010). Utilizing a similar experimental design (i.e., visual habituation), and strikingly similar abstract, animated agents (i.e., a small ball struggling to climb a steep hill) studies find that, around their first birthday, infants’ expectations of and preferences for responsive versus unresponsive caregivers reflect multiple distinct patterns of expectations rooted in personal caregiving experiences.

In these studies, infants are habituated to a large “Mommy” ball climbing a steep hill and leaving her “Baby” at the bottom, crying and unable to follow. Despite clear similarities to the previously described studies, infants’ expectations of, and preferences for, responsive versus unresponsive caregivers varied as a function of personal attachment style. Securely attached infants expected the Caregiver to return to the Baby, while insecurely attached infants expected the Caregiver to ignore the distressed Baby (Johnson et al., 2007; Study 1, Johnson et al., 2010). When the infants were subsequently presented with a video of the Baby alternately approaching a responsive versus unresponsive Mommy, securely attached infants expected the Baby to prefer the responsive Mommy whereas insecurely attached infants expected the Baby to prefer the unresponsive Mommy (Study 3, Johnson et al., 2010). Finally, when infants were shown a partially responsive Mommy (who comes part-way back down the hill to meet the distressed Baby) securely attached infants expected that the Baby would approach the Mommy while insecurely attached infants differed in their expectations based on their unique variety of attachment insecurity. Like securely attached infants, insecure-resistant infants were surprised when the Baby moved further away from the partially responsive Mommy, whereas insecure-avoidant infants were surprised when the Baby approached a partially responsive Mommy (Study 2, Johnson et al., 2010). Together, these findings suggest that relatively stable, early emerging individual differences exert an important influence on the representation and processing of valenced social interactions (Johnson et al., 2007, 2010).

As these two lines of research address common theoretical questions using similar methodologies and stimuli, yet produce different patterns of empirical findings, we are left with an important question regarding how to integrate these results. One explanation is that we only see what we are looking for. It is possible that the helper/hinderer paradigm (e.g., Kuhlmeier et al., 2003; Hamlin et al., 2007) finds universal similarity in reasoning simply because sub-groups were not analyzed. This seems unlikely given that, where counts are available, between 70.5% (Fawcett and Liszkowski, 2012) and 100% (Hamlin et al., 2007) of infants showed similar expectations and preferences in helper/hinderer paradigm yet, only about half of infant samples are securely attached (e.g., 10 out of 21 infants in Johnson et al., 2007; 14 out of 30 infants in Johnson et al., 2010, Study 2; and 20 out of 35 infants in Johnson et al., 2010, Study 3). Moreover, when the responses of the securely and insecurely attached infants were collapsed in the caregiver paradigm, results were not distinguishable from chance (see Johnson et al., 2013). This suggests that the different patterns of results across the two varieties of studies are not simply a reflection of different analytical choices.

An alternative explanation is that these two sets of studies, though superficially similar, actually tap into different underlying representations. Although both sets of studies show a small ball struggling to achieve a goal that is either supported (i.e., when the Helper pushes the Climber up the hill or the Mommy responds to the Baby’s distress) or thwarted (i.e., when the Hinderer pushes the Climber down the hill or the Mommy ignores the Baby’s distress), the two sets of studies may require the attribution of different varieties, or at least complexities, of goals leading to differences in subsequent representations and expectations. In the helper/hinderer studies (e.g., Kuhlmeier et al., 2003), one must represent the Climber’s instrumental goal (i.e., “get up the hill”) in order to interpret and evaluate the subsequent social interactions (i.e., helping versus hindering). In contrast, in the caregiving paradigm (e.g., Johnson et al., 2007), one must represent the Baby’s social-emotional goal (i.e., “get to Mommy”) in order to interpret and evaluate the subsequent social interaction (i.e., responsive versus unresponsive caregiving). Thus, the similar social expectations assessed at test may rely on different initial goal representations and it is this initial willingness or ability to represent the target ball’s goal that may affect the consistency with which social reasoning occurs.

Although both lines of research aim to understand how individuals reason about agents in their environment, it is possible that the different patterns of results reflect important asymmetries in the way individual differences influence our representations of, and expectations about, the goals pursued by others (see also, Johnson et al., 2013) with one set of studies (those employing the helper/hinderer paradigm) relying on the ability to first represent the instrumental goal of an agent (the Climber) acting on an object (the hill) and the other (those employing the caregiver paradigm) relying on the ability to represent the social-emotional goal of an agent (the Baby) acting on another agent (the Mommy; see Spelke, 2014, for a similar distinction). In other words, these two varieties of stimuli may require that participants first represent two different types of goals (i.e., instrumental versus social-emotional) before they can reason about the subsequent social interactions and it is at the level of goal representation that the participants may vary.

### Reconciling Differences

The present research attempts to understand why there appears to be both universal similarities and individual differences in the way individuals reason about those who respond positively versus negatively to others’ unfulfilled goals. By utilizing methods and theory from developmental, social, and cognitive psychology we will examine the extent to which these apparent differences can be understood by examining the types of goals individuals are initially representing when observing these abstract, stylized, animated interactions. Critically, because human infants are limited in the types of responses they can provide, and previous research suggests that there is likely continuity in the way these videos are perceived across the lifespan (see Kuhlmeier et al., 2003), we will examine this question in a much older participant population, namely university undergraduates. In a series of three experiments, utilizing both free-response and eye tracking methodologies, we will examine how attachment security affects the way university undergraduates represent and discuss two varieties of incomplete goals: instrumental need (e.g., agents acting on objects; e.g., Kuhlmeier et al., 2003) and social-emotional distress (e.g., agents acting with agents; e.g., Johnson et al., 2010). In doing so, we aim to demonstrate how the observation of apparent contradictions can help us to develop a more nuanced understanding of the nature of social reasoning.

#### Attachment Security

Early experiences within the caregiver–child dyad are thought to result in internal working models of relationships that organize and bias subsequent social-emotional processing (Bowlby, 1969/1982). Individuals who are securely attached readily approach relationship partners, openly share their needs, and expect that close others will accept and respond appropriately to their distress. In contrast, individuals who are insecurely attached typically avoid or resist their relationship partners and expect close others to reject their needs or respond unpredictably (Ainsworth et al., 1978; De Wolff and van Ijzendoorn, 1997; Cassidy and Shaver, 2008). For decades, researchers have examined the influence of these internal working models of attachment on individuals’ social development, demonstrating that from infancy to adulthood, early experiences in the caregiver–child dyad exert reliable and robust influences on social processing, representation, and behavior (e.g., Main et al., 1985; Dykas and Cassidy, 2011).

Though it is clear that variability in internal working models of attachment affect a number of social-emotional outcomes, what is less clear is where these differences originate. Historically, researchers have examined the link between objective differences in caregiving and attachment security. From this perspective, researchers have found that infants who receive responsive caregiving are considerably more likely to be securely attached than infants who do not (De Wolff and van Ijzendoorn, 1997). Yet, despite the reliability of this finding, variability in the quality of parenting received only accounts for a minority of the observed variance in attachment security. It has recently been proposed that the objective quality of the parenting that the infant receives is less important than the infant’s subjective construal of their experiences (Johnson and Chen, 2011). Specifically, individual differences in attachment can be thought to result not from *objective* differences in social emotional experiences, but from the way these experiences are *subjectively* construed as a function of individual differences in oxytocin receptor (OXTR) genes. In human infants, it appears as though variability in OXTR influences *both* attention to emotionally salient stimuli and attachment security (Johnson and Chen, 2011), suggesting that stable differences in attachment are not simply related to the experiences one has, but to the manner in which one perceives those experiences. Though both accounts posit that variability in attachment security reflects differences in the experience of early caregiving, they differ in regards to whether it is the care received or the construal of the care that biases subsequent social-emotional information processing.

#### Prosocial Behavior

In a related line of research examining the development of other-oriented behavior, there is growing consensus that humans recognize and respond to a variety of problems experienced by others, ranging from relatively simple, emotion-neutral instrumental needs to relatively complex, highly emotional distress (e.g., Dunfield, 2014; Eisenberg et al., 2015). The ability to respond to each of these different types of problems appears to emerge at different ages (e.g., Dunfield et al., 2011) and develop independently of each other (e.g., Svetlova et al., 2010; Dunfield and Kuhlmeier, 2013; Paulus et al., 2013). Together, these findings have led to the proposal that recognizing instrumental need relies on different underlying representations than recognizing emotional distress (e.g., Warneken and Tomasello, 2009; Svetlova et al., 2010; Dunfield, 2014).

Acting effectively on behalf of another requires the ability to represent the problem that the individual is facing, the ability to recognize the required intervention, and the motivation to help alleviate the problem. Recent research supports this position finding that early helping is dependent on children’s abilities to represent stable, abstract goals in others (Hobbs and Spelke, 2015). Yet not all goals are represented with equal ease. Infants represent action goals such as reaching before they understand more mentalistic goals such as using a point to direct attention (Woodward et al., 2001). Relatedly, when examining the literature on the development of the different types of evaluations that may underlie different varieties of prosocial behavior, the ability to represent and reason about others’ instrumental goals appears to emerge earlier than the ability to reason about others’ emotional distress (see Dunfield, 2014, for a review). Moreover, these two varieties of goal attributions are not only dissociable at the developmental level, but appear to be supported by two distinct neural systems. While the mirror neuron system supports the representation of familiar, frequently executed actions based on low-level behavioral input, the metalizing system appears to support the representation of others’ thoughts and beliefs on the basis of social intelligence (Van Overwalle and Baetens, 2009). Finally, these differences in underlying representations affect the ease with which children respond to others’ needs. Although children begin engaging in instrumental help as early as 14 months (Warneken and Tomasello, 2007), social-emotional helping (i.e., getting another’s attention on behalf of a third-party) develops much later (closer to 3 years) and is less frequent and robust (i.e., 16 out of 32 toddlers helping in social tasks versus 29 out of 32 toddlers helping instrumental tasks, Experiment 1; Beier et al., 2014). Together, it is clear that there is considerable heterogeneity in the ability to represent the problems that others face and that these differences affect when and how individuals act on behalf of others.

Critically, attachment security should not necessarily bias the representation of all goals equally. While securely attached individuals have a positive self-construal and feel confident in their ability to accept others’ needs for closeness, sympathy, and support, insecurely attached individuals typically do not. As such, variations in attachment security should exert a greater influence on tasks that require the interpretation of more emotionally laden social stimuli than less emotional instrumental stimuli (see Dykas and Cassidy, 2011, for a review). Because instrumental needs are based on the ability to reason about agents acting on objects, while social-emotional distress requires the ability and willingness to represent another’s negative emotions and social relationships, the ability and willingness to reason about social emotional distress should be uniquely affected by internal working models of attachment. Thus the apparent contradiction in the developmental literature investigating social reasoning may reflect the fact that representing instrumental need is distinct from representing social-emotional distress and the latter shows more variability because it activates, and is influenced by, the social schema that underlie attachment security (e.g., Johnson et al., 2013). However, because attachment security affects attention to, processing of, and the ability to discuss emotionally laden social stimuli, the mechanism through which attachment security will exert its influence is not presently clear.

### Current Study

In order to better understand variability in social reasoning and provide explanatory insight into the apparent contradiction between universal similarity and individual differences in social cognition, we asked university undergraduates to describe a variety of abstract, animated social interactions that were based on the two original hill stimuli (e.g., Kuhlmeier et al., 2003; Johnson et al., 2007). Specifically, we created three brief videos in which a small yellow ball interacted with a large yellow ball and a hill. To disentangle the role of attachment security on the processing of different types of goals, we systematically varied the interaction between the two balls and the hill in order to afford participants the opportunity to discuss both the instrumental (the ball is trying to get up the hill), and social-emotional (the ball is trying to get the attention of, or in proximity to, a social partner) aspects of the interaction. We predicted that if attachment security differentially biases the processing of instrumental needs versus social-emotional distress, then: (1) both securely and insecurely attached participants will discuss instrumental goals similarly (Study 1A); (2) insecurely attached participants will tend to avoid discussing social goals (Study 1B), particularly when the stimuli are complex or ambiguous (Study 2); and (3) any variability in the tendency to report social goals across the two groups will be associated with an attentional bias that is consistent with the underlying attachment representations (Study 3).

## Study 1A

The goal of Study 1 was to determine if individual differences in attachment security affected participants’ recognition of instrumental need versus social-emotional distress. Across two studies, two groups of participants watched as a small ball struggled to complete either an instrumental “hill” goal (Study 1A) or an emotional “social” goal (Study 1B). In both videos, the small ball was separated from a larger ball. However, the videos varied regarding what the little ball was attempting to do. Specifically, in Study 1A (instrumental), the small ball tries but fails to climb the hill, whereas in Study 1B (social), the small ball tries but fails to get the larger ball’s attention. By presenting two separate groups of participants with the two types of goals independently, we can begin to determine the extent to which attachment security imposes an absolute limit on the processing of social stimuli.

### Method

The Office of Responsible Research Practices at the Ohio State University approved all of the research reported in this manuscript.

#### Participants

Ninety-one undergraduate students (39 female) enrolled in an Introductory Psychology course participated for partial course credit.

#### Measures

**FIGURE 1:**
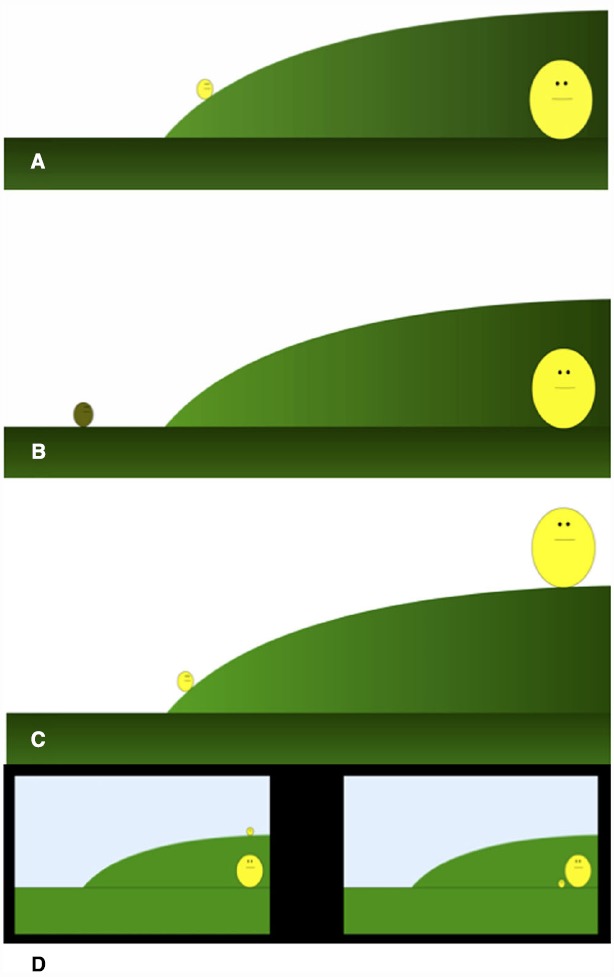
**Schematics of study displays. (A)** Study 1A: hill video; **(B)** Study 1B: social video; **(C)** Study 2: combined video; **(D)** Study 3: outcome scenes.

Participants were shown a brief (20 s) animated video in which a small yellow ball attempts to climb a relatively steep hill while a larger ball looks on (Figure 1A). The small ball makes two attempts at ascent separated by a “sigh” in which the small ball expands and contracts while darkening in color. Both balls had faces but maintained a neutral expression. Following the video, participants were given a small piece of paper and asked to briefly describe what they thought the video was about.

After the participants described the video, they completed the Experiences in Close Relationships questionnaire (ECR; Brennan et al., 1998), which measures attachment security along two dimensions, namely anxiety and avoidance. Attachment anxiety refers to the concern that others will be unavailable in times of need (e.g., “I worry about being abandoned”), while attachment avoidance refers to the tendency to avoid potential pain by keeping others at a distance (e.g., “I feel comfortable sharing my private thoughts and feelings with my partner”). Participants were asked to think about their close relationships in general, without focusing on a specific partner, and rate the extent to which each statement accurately reflects their feelings.

#### Coding

To determine if there were individual differences in the types of goals that the participants attributed, we developed a single coding scheme that we applied consistently across all three free-response studies. First we coded for the presence of any goal directed language. Participants were given a general “goal” code if they used agentive language such as “trying,” “wanting,” “attempting,” or “failing.” Next, we categorized the specific types of goals that the participants identified. Of particular interest was the participants’ tendency to discuss the instrumental (hill) goal and the social (reunion) goal. Hill goals were coded when the participant indicated that the small ball was trying to get up the hill (e.g., “a small circle tried to go up a hill but failed”). Social goals were coded when participants explicitly referred to either a social partner (e.g., a mother, parent, or friend) or a social behavior (e.g., “get attention”) as the small ball’s target. To allow for a more nuanced understanding of the effect of attachment security on the types of goals people represent, these codes were not mutually exclusive. Participants who discussed both goals were given both codes (e.g., “a baby trying to climb the hill to reach his parent”). Some participants discussed the small ball’s behavior in terms of goals that were not related to either the hill or other agent (e.g., “trying to get what you want is not as easy as you think”). These participants received a goal code, but neither of the specific codes. A secondary coder, blind to attachment status and the purpose of the study, coded all of the responses. Agreement was near perfect for goals (96%, κ = 0.92), hills (96%, κ = 0.87), and social goals (94% κ = 0.86).

### Results and Discussion

#### Attachment Classification

To examine whether individual differences in attachment security affects the attribution of goals to others, and to allow comparison to the developmental literature, we created two groups of participants based on their ECR scores (Brennan et al., 1998). Though there are many ways to classify attachment security, we chose to create two groups for our main analysis because the most comparable infant condition found that expectations regarding the Mommy’s behavior (responsive versus unresponsive) differed between securely and insecurely attached infants but did not differ between varieties of insecurely attached infants (see Johnson et al., 2007; Study 1 Johnson et al., 2010). In our sample, the secure group includes individuals who were low on both attachment anxiety and avoidance (*N* = 22, 24.2%, 11 female) and represents individuals who are likely to process both instrumental and social information in an open and relatively accurate manner. In contrast, the insecure group includes participants who are high on one, or both, of the dimensions of attachment insecurity (*N* = 69, 75.8%, 28 female). These individuals are hypothesized to have more negative expectations regarding others’ tendency to seek and accept comfort and are expected to interpret social information in a biased and selective manner. Both attachment anxiety and attachment avoidance were significantly higher in the insecure group than the secure group [anxiety, *t*(89) = 6.72, *p* < 0.001; avoidance, *t*(89) = 6.00, *p* < 0.001].

#### Verbal Reports

**FIGURE 2:**
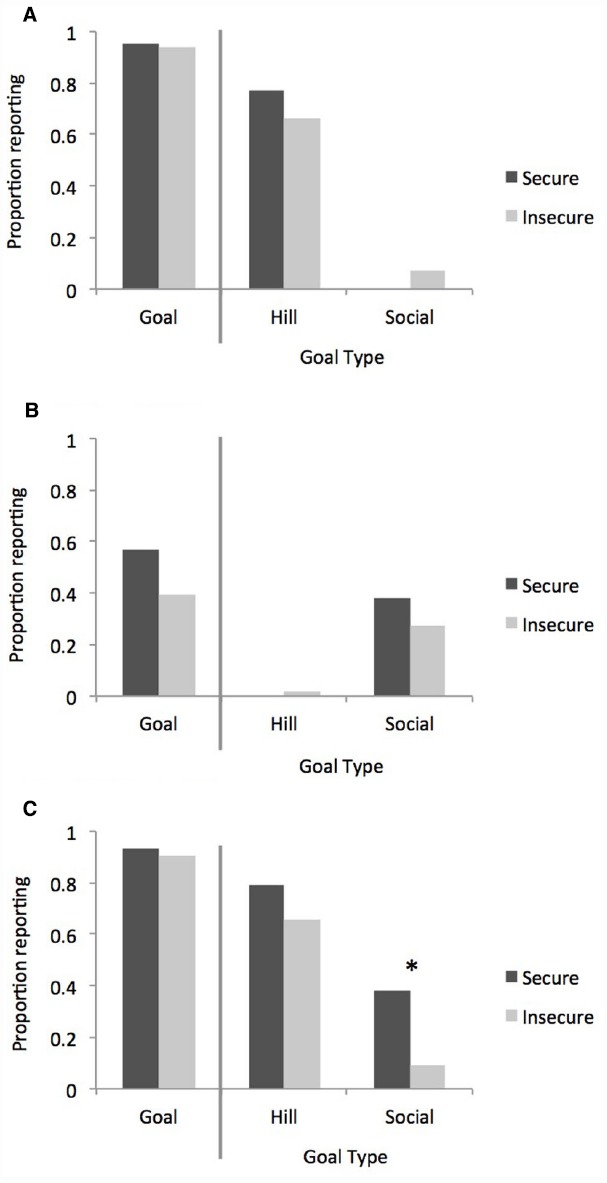
**Results of Studies IA–2.** The bars represent the proportion of participants reporting any goals (left panel), and the specific goals of interest (right panel). **(A)** Study 1A: hill video; **(B)** Study 1B: social video; **(C)** Study 2: combined video. The “*” indicates the difference is significant at *p* < 0.05.

When presented with unambiguous instrumental goals, there were no group-based differences in the tendency to report goals [χ^2^(1, *N* = 91) = 0.05, *p* = 0.82, φ = 0.02], nor in the types of goals reported [hill: χ^2^(1, *N* = 91) = 1.12, *p* = 0.29, φ = 0.11; social: χ^2^(1, *N* = 91) = 1.69, *p* = 0.19, φ = 0.14; Figure 2A]^1^.

Consistent with our predictions, we observed universal similarity in the ability to represent and discuss instrumental goals. When an agent appears to be unsuccessfully acting on an object (in this case a steep hill), attachment security exerts little influence on the ability to represent the underlying goal. Study 1B extends this finding by examining individual differences in the representation and reporting of social goals.

## Study 1B

To determine if variability in the universality of social reasoning is related to differences in the underlying goal representations, particularly when the goals are social, Study 1B presented participants with a video designed to reflect a purely social problem (in this case, a Mommy abandoning her baby).

### Method

#### Participants

Ninety undergraduate students (50 female) enrolled in an Introductory Psychology course, who did not participate in Study 1A, participated for partial course credit. Three additional participants were tested but excluded from analysis for failure to complete all measures.

#### Measures

Study 1B was largely identical to Study 1A with the exception that the video was modified to reflect a single social-emotional goal (Figure 1B). Instead of attempting to climb the hill, the small ball turned to look at the larger ball and then engaged in a series of expansions and contractions, associated with a darkening of color, intended to represent distress. Goals were coded as described in Study 1A. A secondary blind coder coded all reports; agreement was near perfect for goals (100%, κ = 1), hill (98%, κ = 0.98), and social (96%, κ = 0.83). After watching and describing the videos, participants completed the ECR.

### Results and Discussion

#### Attachment Classification

Relative to secure participants (*N* = 21, 23.3%, 12 female), insecure participants (*N* = 69, 76.7%, 38 female) had lower attachment anxiety and avoidance: anxiety [*t*(88) = 5.47, *p* < 0.001] and avoidance [*t*(88) = 6.52, *p* < 0.001].

#### Verbal Reports

Despite being presented with a purely social interaction, there were no attachment related differences in the tendency to report goals [χ^2^(1, *N* = 90) = 2.13, *p* = 0.15, φ = 0.15], nor in the specific goals reported [hill: χ^2^(1, *N* = 90) = 0.31, *p* = 0.58, φ = 0.05; social: χ^2^(1, *N* = 90) = 0.85, *p* = 0.35, φ = 0.10; Figure 2B]^2^.

Study 1B replicates and extends the findings of Study 1A by demonstrating that when presented with pure and unambiguous goals, individuals make the same attributions regardless of goal type or attachment categorization. Though these findings appear to suggest that differences in attachment security do not differentially influence the ability to represent instrumental versus social goals, because social schemas, such as internal working models of attachment, are particularly likely to bias processing when stimuli are complex or ambiguous (e.g., Baldwin, 1992; Johnson et al., 2013) it is possible that separating the two goals and presenting them independently and unambiguously diluted the effect.

Consistent with the proposal that schemas have a greater influence on the representation of ambiguous stimuli, Johnson et al. (2007, 2010) first documented individual differences in social reasoning when both the hill and social goal were presented together. Unlike our pure videos, the original caregiver paradigm showed the Mommy distressing the Baby by climbing up a steep hill, affording both an instrumental (the baby simply cannot get up the hill) and social-emotional (the baby is distressed because it cannot get to its Mommy) problems. Given this design, it is possible that different participants were attending to different aspects of interaction. To address this consideration, and explore the extent to which attachment security affects the interpretation of *complex/ambiguous* problems, we modified our videos to make them more similar to Johnson et al. (2007). Specifically, we created a new video in which both the hill and social goals were equally salient.

## Study 2

Study 2 aimed to determine if individual differences in attachment security affected participants’ recognition of instrumental need versus social-emotional distress in complex scenes. To that end, participants watched a video that included *both* the instrumental “hill” goal of Kuhlmeier et al. (2003), and the social “reunion” goal of Johnson et al. (2010). Because the video was complex and included both an instrumental and social goal, we predicted that although all participants should be able to recognize goal directed behavior, and both groups of participants should be equally likely to discuss the instrumental goal, insecurely attached individuals will avoid reporting the social goal because this video, unlike the pure social video, affords this option.

### Method

#### Participants

Ninety-three undergraduate students (45 female) enrolled in an Introductory Psychology course participated for partial course credit. One additional student participated in the study but failed to complete all the measures and was removed from subsequent analysis.

#### Measures

Largely identical to the previous two studies, the only modification was the content of the videos. Specifically, we moved the large ball from the bottom of the hill to the top thus combining the small ball’s instrumental and social goals (Figure 1C). In order to make both varieties of goals equally salient, and comparable to Studies 1A/B, the small ball attempts to climb the hill once, expands and contracts once, then, at the bottom of the hill, expands and darkens in color, appearing to cry. The larger ball remains motionless at the top of the hill for the duration of the video. Consistent with the previous videos, both balls had faces but maintained a neutral expression. Following the video participants completed the ECR. Again, all reports were coded by a secondary, blind coder and agreement was high (97%, κ = 0.79), hill (94%, κ = 0.84), and social (98%, κ = 0.93).

### Results and Discussion

#### Attachment Classification

Both attachment anxiety and avoidance were lower in the secure group (*N* = 29, 31.2%, 11 female) than the insecure group [*N* = 64, 68.8%, 34 female; anxiety, *t*(91) = 5.74, *p* < 0.001; avoidance, *t*(91) = 5.98, *p* < 0.001].

#### Verbal Reports

Both groups of participants were equally likely to discuss the ball’s behavior in agentive, goal-directed language [χ^2^(1, *N* = 93) = 0.16, *p* = 0.69, φ = 0.04; Figure 2C]. Moreover, both groups were equally likely to recognize and report the instrumental “hill” goal [χ^2^(1, *N* = 93) = 1.78, *p* = 0.18, φ = 0.14]. However, consistent with our hypotheses, the groups differed in their tendency to report the “social” goal [χ^2^(1, *N* = 93) = 10.89, *p* = 0.001, φ = 0.34]^3^; specifically, insecurely attached participants were significantly less likely than securely attached participants to report the Baby’s social goal of reuniting with the Mommy.

To determine whether it was attachment insecurity in general or one of the continuous attachment dimensions in particular that affected participant’s tendency to report the social goal, we conducted a logistic regression with attachment anxiety, avoidance, and their interaction as continuous, independent predictors. The overall model accounted for a significant portion of the variance [χ^2^(3, *N* = 93) = 8.17, *p* = 0.04]; however, none of the independent predictors were significant [*p*’s > 0.154]. Indicating that it is attachment insecurity broadly, as opposed to either of the specific dimensions that influenced the participants’ tendency to report the social goal.

Study 2 examined the extent to which individual differences in attachment security affected the representation of two varieties of goals that varied in their social-emotional content. Differences in attachment security exerted a greater influence on the processing of social-emotional than instrumental goals, but only when the two types of goals were presented together. This result appears consistent with past research suggesting that insecure attachment categorically biases social representations irrespective of the nature of the insecurity (i.e., avoidance or anxiety; see Johnson et al., 2007, for a similar result). Moreover, these findings support the proposal that an individual’s pre-existing relationship schema exerts a greater influence on representations when evaluating ambiguous, as opposed to clear, stimuli (e.g., Baldwin, 1992).

Though Study 1B rules out the possibility that these results reflect a general unwillingness of insecurely attached individual to discuss social emotional needs, it is not clear from these descriptions whether attachment security is biasing the way participants are attending to and representing the interaction or simply the way participants are discussing the interaction. Study 3 uses eye-tracking methodology to determine the extent to which the differential discussion of social-emotional goals observed in Study 2 is driven by differences in underlying attention and representation.

## Study 3

Study 3 presented participants with the same stimuli as Study 2, but instead of having them provide a written description, we presented two outcomes intended to represent the successful completion of either the hill or social goal. Because infants have limited verbal abilities, methodologies for assessing mental representations that do not require verbal responses have become an invaluable tool to developmental psychologists (see Oakes, 2010, for a comprehensive review of this methodology). Though visual attention varies greatly across the lifespan (Colombo, 2001), gaze duration has previously been used in adult populations to examine attention to, and expectations of, similarly social stimuli (e.g., Guastella et al., 2008). Further, although it is less common to utilize looking time methodologies to assess the social cognitive representations of adults, doing so allows for a more direct comparison to the developmental literature that motivated the current research. Following the logic of infant looking time designs (e.g., Spelke, 1985), we expect that participants who have an expectation regarding the ball’s goal will show greater attention to, and spend more time looking at, the outcome they find relatively unexpected.

### Method

#### Participants

Two-hundred and twenty-nine undergraduate students (126 female) received partial course credit for participation. Two additional students participated in the study but failed to complete the ECR and were removed from subsequent analysis. Participants whose Tobii capture rate was less than 75% were also excluded from further analysis. The final sample included 192 participants (103 females).

#### Measures/Procedure

Participants were 5-point calibrated on a Tobii T60 XL eye tracker. Once calibrated, participants watched the complex video from Study 2. Participants then saw a flashing central fixation point followed by two static outcomes presented simultaneously (Figure 1D). Because the complex video affords two accurate goal attributions (i.e., hill *and* social), we created two resolution scenes that dissociated these two outcomes. In both scenes the large ball was moved to the bottom of the hill, however, the location of the small ball varied. In the *hill* outcome, the small ball was seated atop the hill, physically separated from the large ball. In contrast, in the *social* outcome, the small ball was at the bottom of the hill beside the large ball. areas of interest (AOIs) were created around both of the outcomes and total fixation duration during the first 5 s of presentation was analyzed. Direction of motion (left versus right) and location of outcome (left versus right) were counterbalanced between participants. Following the eye-tracking portion of the study participants completed the ECR.

### Results and Discussion

#### Attachment Classification

Consistent with the previous studies, participants were split into a secure (*N* = 67, 34.9%, Female = 34) and insecure group (*N* = 125, 65.1%, Female = 68). The securely attached group had significantly lower anxiety and avoidance than the insecure group [anxiety, *t*(190) = 9.26, *p* < 0.001; avoidance, *t*(190) = 9.60, *p* < 0.001].

No main effects or interactions of gender were observed; thus it was removed from subsequent analyses. A 2 (outcome: hill, mom) × 2 (security: secure, insecure) × 2 (motion: left, right) × 2 (location: left hill, left mom) mixed model analysis of variance was conducted to determine if the two groups of participants differentially attended to the two outcomes. There was a significant main effect of outcome [*F*(1,184) = 13.47, *p* < 0.001, ${\text{η}}_{p}^{2}$ = 0.07], and location [*F*(1,184) = 5.286, *p* < 0.023, ${\text{η}}_{p}^{2}$ = 0.03], and an interaction between outcome, motion, and location [*F*(1,184) = 22.75, *p* < 0.001, ${\text{η}}_{p}^{2}$ = 0.11].

**FIGURE 3:**
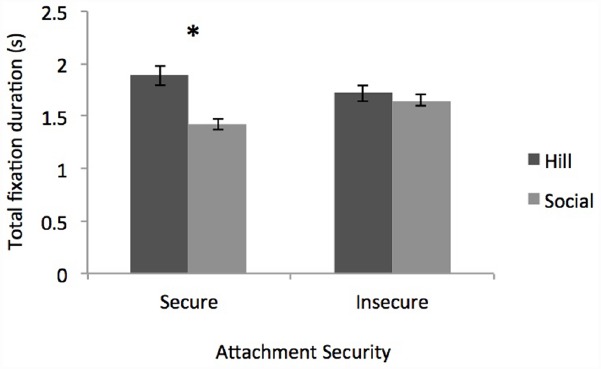
**Participant’s total fixation duration to the instrumental (hill) versus social (reunion) outcome by attachment security.** The “*” indicates the difference is significant at *p* < 0.05.

Of particular interest to our research question was the effect of attachment security on attention to the two outcomes. As predicted by an attentional bias account, we found a significant interaction between security and outcome [*F*(1,184) = 6.795, *p* < 0.01, ${\text{η}}_{p}^{2}$ = 0.04; Figure 3]. Securely attached participants spent significantly more time looking at the hill outcome (*M* = 1.89, SD = 0.77) than the social outcome (*M* = 1.42, SD = 0.63) whereas, the insecurely attached participants looked equally long at both the hill (*M* = 1.72, SD = 0.58) and social outcomes (*M* = 1.65, SD = 0.57). Finally, security and outcome interacted with location [*F*(1,184) = 6.22, *p* < 0.01, ${\text{η}}_{p}^{2}$ = 0.03] such that, securely attached participants showed a main effect of outcome [*F*(1,65) = 10.47, *p* = 0.002, ${\text{η}}_{p}^{2}$ = 0.14] regardless of location [*F*(1,65) = 3.12, *p* < 0.08, ${\text{η}}_{p}^{2}$ = 0.05]. In contrast, insecurely attached participants showed a marginal interaction between outcome and location [*F*(1,123) = 3.75, *p* < 0.06, ${\text{η}}_{p}^{2}$ = 0.03] but no main effect of outcome [*F*(1,123) = 0.92, *p* = 0.34, ${\text{η}}_{p}^{2}$ = 0.007]^4^. Simply put, though securly attached participants tended to look longer to the hill outcome regardless of where it was located, insecurely attached participants tended to look longer at whichever outcome that was closer to the ball’s initial movement, regardless of its content. A Wilcoxon Signed-rank test revealed a significant effect of attachment security on preferred outcome (*z* = –2.12, *p* = 0.034), suggesting that the pattern of results is not simply a function of averaging but instead holds across individual group members.

Taken together, this pattern of results suggests that securely attached participants have automatic, robust implicit expectations regarding the ball’s ultimate goal that supersede task demands. Consistent with, though stronger than, the verbal responses observed in Study 2, securely attached participants appear to represent the hill as a means of achieving the overarching social goal. Securely attached participants devoted more attention (operationalized as longer looking) to the hill outcome, where the ball has successfully overcome a physical barrier but remains separated from its social partner, than to the social outcome, where the ball remains at the bottom of the hill but is reunited with its social partner, suggesting that participants found the instrumental outcome less expected and requiring of more attention. In contrast, insecurely attached participants did not differentiate their attention to either of the outcomes suggesting that they are either not automatically prioritizing social over instrumental goals, or are doing so in a manner that is weak and quickly overwhelmed by the surface characteristics of the stimuli. Insecurely attached participants did not appear to have a strong expectation regarding either resolution. Together with Study 2, these results support the proposal that variability in attachment security can influence the way we represent others’ goals. When participants process complex social interactions that afford a number of different construals, the ease with which an individual approaches and interacts with their social environment can bias the representation of social-emotional goals particularly when the social goals are ambiguous and paired with a less emotionally evocative instrumental goal.

## General Discussion

The overarching goal of the present research was to begin to address the question of why some early social reasoning appears universal, while some shows marked individual differences. Specifically, using both free-response and eye-tracking methodologies, we attempted to bridge two related domains of literature examining attachment security and other-oriented behavior in order to determine if this apparent contradiction could serve as a starting point for future research.

Across a series of three studies we demonstrate that the individual difference variable of attachment security affects the representation of instrumental needs differently than social-emotional distress (Studies 2 and 3). However, this was only the case when the stimuli were complex and afforded multiple potential interpretations (Studies 1A, B). Together these results suggest that attempting to understand and integrate divergent findings within a single theoretical framework can lead to more nuanced understanding.

Though these studies approach the question of social reasoning from a novel perspective, the findings are largely consistent with existing literature. As predicted by attachment theory we observed an influence of attachment security on the representation of social-emotional stimuli (Dykas and Cassidy, 2011), particularly when the stimuli were complex and afforded multiple construals (Baldwin, 1992). In addition, these findings are consistent with a growing body of literature examining the social cognitive constraints on early other-oriented behaviors; particularly that the ability to recognize and respond to instrumental needs emerges prior to, and independent from, the ability to respond to emotional distress (see Dunfield, 2014, for a review). Further, these results may help to explain why the ability to provide instrumental help appears more robust, and earlier emerging, than the ability to offer social help (Beier et al., 2014). Finally, these results are consistent with the finding that infants appear to universally evaluate helpers positively and hinderers negatively (e.g., Kuhlmeier et al., 2003; Hamlin et al., 2007), while showing individual differences in their expectations of responsive versus unresponsive caregivers (Johnson et al., 2007, 2010). Indeed, by considering both the underlying task demands and bodies of related research, we can gain insight and support for the perspective that attributing instrumental goals to agents acting on objects requires different underlying representations than attributing social emotional goals to agents acting on other agents (e.g., Spelke, 2014).

By taking a broad approach to social-cognitive development, and attempting to integrate a diversity of findings into a single theoretical account, we demonstrate an important role for examining how an individual difference variable, such as attachment security, can influence *both* similarities and differences in social reasoning across individuals. Importantly, this work represents a first step toward integrating two approaches that have previously been largely pursued independently. While inspired by developmental theory and research, these studies examined university undergraduates, leaving open the question of when and how early social experiences influence cognitive development; however, based on existing findings (e.g., Johnson et al., 2007, 2010) it appears that the reciprocal relations emerge early.

Though these findings are consistent with previous research, the mechanisms underlying these differences are unclear. Based on these studies it is not currently possible to determine whether it is the content or complexity of the underlying representation that drives the observed differences. Future work is required to determine how these different goal construals interact with social evaluations in order to support behavioral outcomes. For example, it is possible that the infants in the initial caregiver studies (Johnson et al., 2007, 2010) were differentially evaluating the responsive versus unresponsive caregiver because they differed in the initial goal representation (i.e., some infants attended to the Baby’s distress while others were attending to the Mommy’s climb). It is also possible that the goals were construed similarly (i.e., reunion), however the participants differed, as proposed, in the type of caregiving responses they expected.

The goal of this paper was to examine more directly the apparent contradiction between research supporting universal similarities and individual differences in social reasoning. Although these two perspectives were often examined separately by researchers interested in either innate, early-emerging, universal components of cognition, or researchers interested in variable social outcomes affected by experience, there is a growing move to bring these two perspectives back together (Olson and Dweck, 2008, 2009). While this recombination may lead to the appearance of contradiction and inconsistency, we have demonstrated that by addressing the conflict head on, and using points of tension as starting points for further investigation, we can work toward a more nuanced and accurate understanding of the nature of human social cognition.

### Conflict of Interest Statement

The authors declare that the research was conducted in the absence of any commercial or financial relationships that could be construed as a potential conflict of interest.

## Footnotes

1 We conducted the same analyses treating the three varieties of attachment insecurity separately and observe the identical pattern of results: Goals: χ^2^(3, *N* = 91) = 1.2, *p* = 0.75, φ = 0.11; Hill: χ^2^(3, *N* = 91) = 3.90, *p* = 0.27, φ = 0.21; Social: χ^2^(3, *N* = 91) = 3.15, *p* = 0.37, φ = 0.19.

2 Again, the pattern of results remains the same when the three varieties of attachment insecurity are treated as separate groups: Goals: χ^2^(3, *N* = 90) = 2.31, *p* = 0.51, φ = 0.16; Hill: χ^2^(3, *N* = 90) = 3.32, *p* = 0.34, φ = 0.19; Social: χ^2^(3, *N* = 90) = 1.25, *p* = 0.74, φ = 0.12.

3 We analyze the three varieties of attachment insecurity separately the pattern of results is identical: Goals: χ^2^(3, *N* = 93) = 1.50, *p* = 0.68, φ = 0.13; Hill: χ^2^(3, *N* = 93) = 3.49, *p* = 0.32, φ = 0.19; Social: χ^2^(3, *N* = 93) = 11.33, *p* = 0.01, φ = 0.35.

4 The pattern of results largely replicates when attachment insecurity is separated into three groups, however the interaction between ECR and outcome falls from significant to trending [*F*(1,176) = 2.40, *p* < 0.07, ${\text{η}}_{p}^{2}$ = 0.04].
